# Supplementary material for: Thermal-comfort evaluation of and plan for public space of Maling Village, Henan, China
Source: PLoS One. 2021 Sep 20;16(9):e0256439. doi: 10.1371/journal.pone.0256439 (PMC8452085; doi:10.1371/journal.pone.0256439)
Supplement: S1 File — (DOCX) [file pone.0256439.s001.docx]

**S1 File**

**Outdoor Thermal comfort questionnaire**

Date: / /____ Time: __________

**1 Gender**

Male
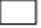
 Female
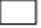


Height: _________ Weight: _________

**2 What is your age group range?**

18-29 years

30-40 years

41-50 years

51-60 years

61-64 years

1. years and older

**3 What are you wearing now? (multiple choice)**

Pale colors Neutral Dark colors

Upper: Sleeveless vest Short-sleeve T-shirt

Long- sleeve T-shirt Long- sleeve blouse Sweater

Bottom: Short shorts Straight trousers (thin) long skirt

Short skirt Other_______

4 **Location:** Space with fitness facilities Unsheltered square

Spaces with shading from trees Unshaded lawns Green park spaces Pavilions Areas with shading from buildings Roads

**5 Please describe your overall comfort level?**

**（Note:** Please vote according to your actual situation at this time）

Very comfortable Slightly comfortable Neutral Uncomfortable Very uncomfortable

**6 How do you feel at this moment?**

**
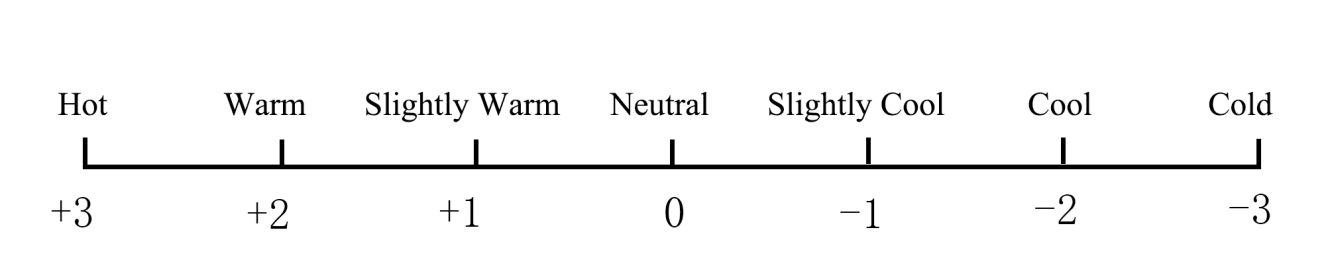
**

**7 Your current activity?**

Standing (chatting, playing mobile phone, enjoy scenery, etc.)

Seating (chatting, playing mobile phone, reading, etc.)

Strolling

Low-intensity exercising (brisk walking, looking after children, walk the dog, etc.)

Medium-intensity exercising (Jogging, etc.)

High- intensity exercising (ball games, square dancing, etc.)

**8 How long have you been here?**

＜15 min 15-30 min 30-60 min ＞60 min

**9 What time do you like to come here during the day?**

6:00-8:00 8:00-10:00 10:00-12:00 12:00-14:00 14:00-16:00 16:00-18:00 18:00-20:00 20:00-21:00

**10 Which meteorological parameters do you think have the greatest impact on thermal comfort?** **(select two of them)**

Air temperature Relative humidity Wind speed Solar radiation

**11 You will be more comfortable if the environment (single choice for each)**

Air temperature: Higher Unchanged Lower

Relative humidity: Damper Unchanged Drier

Wind speed: Stronger Unchanged Weaker

Solar radiation： Stronger Unchanged Weaker

**室外热舒适问卷调查**

日期: / /____ 时间: __________

**1 性别**

男
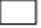
 女
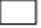


身高: _________ 体重: _________

**2 您处于哪个年龄阶段?**

18-29 岁

30-40 岁

41-50 岁

51-60 岁

61-64 岁

65岁及以上

**3 您现在穿什么? (多选)**

白色 中性色 黑色

**上部:**  无袖背心 短袖T恤 长袖T恤

长袖衬衫 毛衣

**下部:**  超短裤 直裤（薄） 长裙

短裙 其他_______

4**位置:** 有健身设施的地方 无遮挡的广场 遮阳树下

无遮阳的草坪 公园绿地 凉亭

建筑物的阴影区 道路

**5 请描述一下您的整体舒适度？**

（注：请根据本次实际情况投票）

非常舒适 略微舒适 适中的 不舒服 很不舒服

**6 此时此刻你感觉如何?**

**
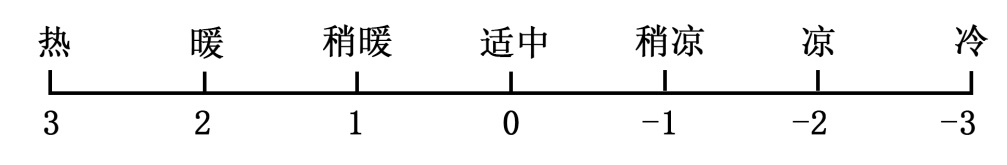
**

**7 您目前的活动?**

站立 (聊天, 玩手机, 欣赏风景等)

坐着 (闲聊, 玩手机, 阅读等)

散步

低强度运动 快步走, 照看孩子, 遛狗等)

中强度运动 (慢跑等)

高强度运动 (球类运动,广场舞等)

**8 您在这里多久了?**

＜15 分钟 15-30 分钟 30-60分钟 ＞60分钟

**9 在一天中，您喜欢什么时候来这里？**

6:00-8:00 8:00-10:00 10:00-12:00 12:00-14:00

14:00-16:00 16:00-18:00 18:00-20:00 20:00-21:00

**10 您认为哪些气象参数对热舒适的影响最大？** **(选择其中的两个)**

空气温度 相对湿度 风速 太阳辐射

**11 如果环境是……你会感觉更舒适（每类选择一个）**

空气温度: 更高 不变 更低

相对湿度: 更潮湿 不变 更干

风速: 更强 不变 更弱

太阳辐射： 更强 不变 更弱
