# Supplementary material for: Thermal-comfort evaluation of and plan for public space of Maling Village, Henan, China
Source: PLoS One. 2021 Sep 20;16(9):e0256439. doi: 10.1371/journal.pone.0256439 (PMC8452085; doi:10.1371/journal.pone.0256439)
Supplement: S2 File — (DOCX) [file pone.0256439.s002.docx]

**Data of subjective evaluation in this study**

**Table1. Evaluation of the influence of certain characteristics on subjective comfort**

| **Parameter** | **Number of people** |
| --- | --- |
| Air temperature | 203 |
| Relative humidity | 28 |
| Wind speed | 82 |
| Solar radiation | 99 |

**Table2. Overall comfort votes for different spaces**

| **Location**  **Overall comfort votes** | **Space with fitness facilities** | **Unsheltered square** | **Shading from trees** | **Unshaded lawns** | **Green spaces in parks** | **Pavilions** | **Areas with shading from buildings** | **Roads** |
| --- | --- | --- | --- | --- | --- | --- | --- | --- |
| **Very uncomfortable** | 2 | 2 | 0 | 2 | 0 | 0 | 0 | 5 |
| **Uncomfortable** | 2 | 15 | 1 | 4 | 0 | 2 | 2 | 18 |
| **Neutral** | 10 | 12 | 8 | 20 | 4 | 10 | 5 | 10 |
| **Slightly comfortable** | 25 | 8 | 20 | 12 | 25 | 30 | 20 | 3 |
| **Very comfortable** | 5 | 2 | 60 | 3 | 30 | 20 | 12 | 3 |

**Table3. Thermal sensation votes for different spaces**

| **Location**  **Thermal sensation votes** | **Space with fitness facilities** | **Unsheltered square** | **Shading from trees** | **Unshaded lawns** | **Green spaces in parks** | **Pavilions** | **Areas with shading from buildings** | **Roads** |
| --- | --- | --- | --- | --- | --- | --- | --- | --- |
| **-1(slightly cool)** | 0 | 0 | 1 | 0 | 2 | 0 | 5 | 0 |
| **0 (neutral)** | 2 | 4 | 50 | 18 | 30 | 28 | 20 | 2 |
| **1(slightly warm)** | 8 | 12 | 12 | 25 | 5 | 7 | 5 | 10 |
| **2(warm)** | 15 | 25 | 5 | 8 | 4 | 5 | 4 | 38 |
| **3(hot)** | 5 | 12 | 2 | 3 | 2 | 4 | 2 | 32 |

**Table4. Physiological equivalent temperature and Mean thermal sensation vote**

| **Physiological equivalent temperature** | **Mean thermal sensation vote** |
| --- | --- |
| 25.2 | 0.4 |
| 28.3 | 0.8 |
| 29.1 | 0.5 |
| 30.2 | 0.7 |
| 31.2 | 1.5 |
| 31.8 | 1.2 |
| 32.4 | 1.0 |
| 33.0 | 1.5 |
| 34.0 | 1.6 |
| 35.0 | 1.7 |
| 36.0 | 1.5 |
| 37.0 | 1.9 |
| 38.0 | 2.1 |
