# Supplementary material for: Thermal-comfort evaluation of and plan for public space of Maling Village, Henan, China
Source: PLoS One. 2021 Sep 20;16(9):e0256439. doi: 10.1371/journal.pone.0256439 (PMC8452085; doi:10.1371/journal.pone.0256439)
Supplement: Attachment — Submitted filename: Thermal-Comfort Evaluation of and Plan for Public Space (AutoRecovered).docx [file pone.0256439.s004.docx]

Thermal-Comfort Evaluation of and Plan for Public Space of Maling Village, Henan, China

**Qindong Fan ^1^,Fengtian Du ^2,*^, Hu Li ^1^ , Chenming Zhang ^1^**

1	School of Architecture, North China University of Water Resources and Electric Power, Zhengzhou, China

2	School of Art and Design, North China University of Water Resources and Electric Power, Zhengzhou, China

*****	Correspondence : dufengtian@126.com

# Abstract

The thermal conditions of a public space play an important role in villagers’ life. Previously published studies of thermal comfort mainly focused on the measurement and evaluation of thermal comfort, and few studies on thermal comfort planning and construction process. The study was carried out in Maling Village during the summer, integrating a questionnaire survey and a field measurement (air temperature, relative humidity, black globe temperature, and wind speed of three typical public spaces）was measured. Subsequently, the village’s microclimate environment was simulated with ArcGIS 10.6 and ENVI-met. The results indicate that during the summer, the influences of temperature, solar radiation, wind speed, and relative humidity on the subjective comfort conditions of the outdoor environment gradually decreased. The shapes of the public spaces, the configuration of plants, the nature of underlying surfaces, the layouts of buildings, the directions of streets, and the common outdoor activities of the villagers could significantly affect the thermal comfort level. Finally, based on the analysis of the results, this study put forward the planning and construction methods of the thermal comfort environment of the village outdoor space.

**Keywords:** thermal comfort, village, public space, evaluation, planning

# 1. Introduction

Approximately half the world's population lives in rural areas [1], the outdoor spaces of which have great functional importance in the residents’ lives. Rapid urbanisation has disturbed the rural outdoor environment [2]. In the past, rural planning has primarily considered spatial layout, culture, ecology, and other village functions [3, 4]. As such, the relationship between a village’s spatial form and microclimate has most neglected. As a result, the outdoor spaces of villages are generally characterised by relatively poor thermal comfort. This in turn has lowered villagers’ willingness to perform outdoor activities – a reluctance that has negatively affected their well-being [5, 6]. Nevertheless, village outdoor spaces should be constructed in the same way as urban outdoor spaces that are attractive and comfortable [7]. Therefore, thermal conditions of villages’ outdoor environments have been attracting increasing attention [8, 9].

Over the past few years, the interest in outdoor thermal environments has increased, and thermal comfort (as a direct reflection of human thermal feelings towards environments) has rapidly gained importance as a research topic [10]. Studies of thermal comfort conditions can be traced back to the 1930s [11]. Currently, thermal comfort is defined as the ‘condition of mind that expresses satisfaction with the thermal environment and can be assessed by subjective evaluation’ [12]. There are three aspects of factors that affect outdoor thermal comfort: the physical, personal, and social. Physical factors include air temperature, relative humidity, solar radiation, and wind speed [13]. Personal and social factors include a resident’s gender, age, metabolism, residence time, emotional status, cultural background, and environmental attitude [14-16]. Many researchers have investigated outdoor thermal comfort and its related factors by field experiments [17-23] and numerical simulation [24-29]. Field experiments measure outdoor environments parameters, such as air temperature, relative humidity, black globe temperature, and wind speed, and subjective questionnaire surveys, which were conducted near the monitoring area [30, 31]. The purpose of field measurements was to determine the thermal conditions that are comfortable or acceptable for local residents, understand the residents’ perceptions of thermal comfort [32, 33], and provide a valuable reference for the government [34, 35]. Field measurements are currently considered the key evaluation method for outdoor thermal comfort characteristics [36, 37].

Meanwhile, numerical simulation is another important method for outdoor thermal environment analysis, in which ENVI-met software has been one of the most widely used platforms [38, 39]. ENVI-met is suitable for small- and medium-scale microclimate simulations; it has been used in relevant case studies of hot-summer-cold-winter regions [48], hot-summer-warm-winter regions [38, 49], temperate regions [50, 51], cold regions [52], hot and arid regions [53, 54], and tropical regions [55, 56]. Furthermore, it has been applied to many different outdoor microclimate spaces, such as streets [57], campus areas [34], parks [58], and residential communities [59].

The most common thermal-comfort evaluation indexes are the standard effective temperature (SET), predicted mean vote (PMV), PET, universal thermal climate index (UTCI), mean radiant temperature (T_mrt_), wet bulb globe temperature (WBGT), and discomfort index (DI) [60]. The PMV is determined by the stable indoor environment [61], and a seven-point scale is often used to predict the average thermal response of many people in indoor thermal-comfort evaluations [62]. The UTCI is an easy-to-determine body heat index, which can be applied to different seasons, climates, and dimensions [63, 64]. Furthermore, the WBGT, SET, T_mrt_, and DI have been widely used to assess the outdoor thermal comfort characteristics of different climate zones [65-67]. The PET, which is more sensitive than UTCI to changes in human thermal response, is a widely studied parameter in thermal-comfort research on weather forecasting, urban planning and design [67, 68], and the outdoor evaluation of complex environments in different climate zones [69, 70]. The calculation of PET is also more accurate than that of UTCI [71].

The majority of published studies on thermal comfort focused on urban spaces and building interior [72-78], and the research results often limited to the measurement and evaluation of thermal comfort indexes. In this study, the ArcGIS 10.6 platform, ENVI-met 4.4.5 software, and PET index were used to evaluate the thermal comfort characteristics of outdoor public spaces (i.e., streets, squares, and green space) in Maling Village, Henan, China. Based on thermal-comfort simulations, a specific village thermal-comfort planning scheme and construction process are proposed.

# 2. Materials and Methods

2.1 Ethics statement

The field studies were conducted at a village public open space in Changdai Town, Mengjin County, Luoyang City, Henan Province, China (Fig 1a). Thus, with the support of local town government and village committee, we conducted a questionnaire survey and field measurement. The experiments conducted in this study did not involve endangered or protected species. For a detailed explanation, please see the Supporting Information.

2.2. Study area

The study area is in a typical hilly village in the west of Changdai Town, Mengjin County, Luoyang City, Henan Province, China (Fig 1a). It belongs to the warm-temperate (Dwa) [79] climate zone, which is hot and rainy during the summer and cold and dry during the winter. The meteorological statistics from 2010 to 2019 in Fig. 1(b) presents the meteorological data from the China Meteorological Information Centre [80]. The land use data of Maling Village originated from China's third land survey, which was conducted in 2018 (Fig. 1c).

Maling Village is the central village; the highway (Xiaolangdi special line) passes through it. The other three villages under the jurisdiction of Maling are Xiamaling, Donggou, and Yangjiazui (Fig. 1a). Maling Village has a total area of 51 ha and a population of 1419. Agriculture is a crucial sector of Maling Village. Furthermore, the village has well-equipped public service facilities, and residents can perform a variety of outdoor activities in street spaces, squares and many other green spaces. The average building height is relatively low (one to three storeys high).

**Fig 1. (a) Geographic location of the study area. (b) Monthly mean/maximal/minimal air temperature and mean relative humidity in Mengjin County from 2010 to 2019. (c) Land use map.**

2.3. Selection of measurement points

Three typical public spaces (squares, streets, and green space) in Maling Village were chosen as study areas based on the frequency of villager visits. A total of 15 measurement points were selected. Seven measurement points were on roads (the measurement points were located on the central axes of roads with buildings on both sides), including four east–west streets and three north–south streets. In addition, four measurement points were in the squares and green spaces of the village, respectively. The specific positions of the measurement points are shown in Fig 2. Moreover, Table 1 lists the current spatial measurement points.

The study was performed during the summer (from June 12^th^ to 17^th^, 2020); the period covered weekends and working days; data from days with bad weather (e.g., rain) were excluded. Measurements were made every two hours from 6:00 until 20:00. The measurement time at each sampling point was 5 min.

**Fig 2.** **Distribution of measurement points.**

**Table 1. Different types of spatial measurement points.**

| **Square** | | | | | |
| --- | --- | --- | --- | --- | --- |
| Description |  | |  |  |  |
|  | Basketball square (Point 1), located across the road from the village committee. | | Pavilion square (Point 2), located on the north side of the East–West Street of Market Road. | Triangle square (Point 4), located on the east side of the North–South Street of Market Road. | Fitness square (Point 14), located on the south side of Photovoltaic Road. |
| **East–West Street** | | | | | |
| Description | |  |  |  |  |
|  |  | East–West Street  of Market Road (Point 5) | Maling 5^th^ Group Road (Point 8) | Photovoltaic Road (Point 11) | West Street of  primary school (Point 12) |
| **North–South Street** | | | | | |
| Description | |  |  |  |  |
|  |  | North–South  Street of Market Road (Point 3) | Park Road (Point 7) | North–South Street of School Road (Point 15) |  |
| **Greenland** | | | | | |
| Description | |  |  |  |  |
|  |  | Green space on west side of the village (Point 6) | Green space on east side of Park Road (Point 9) | Green space on west side of Park Road (Point 10) | Green space on south side of the village (Point 13) |

2.4. Methods

The data sources included subjective and objective evaluations; the subjective data included the residents’ evaluations, which were based on the residents’ perceptions and experiences of the village's thermal environment. The objective data were measured with instruments.

### 2.4.1. Subjective evaluation

The subjective evaluation is comprised of a questionnaire sampling survey on the villagers’ comfort and satisfaction regarding the outdoor public space. First, the questionnaire was used to collect relevant data (such as..). Second, the temporal distribution of villagers’ outdoor activities and the influence of environmental parameters (such as…) were studied, and the overall thermal comfort and thermal sensation characteristics of different spaces were evaluated. Finally, the relationship between the mean thermal-sensation vote (TSV) and PET was analysed to provide thermal-comfort planning based on the residents’ subjective feelings.

(1) Evaluation of overall outdoor thermal comfort and thermal sensation characteristics

The questionnaire study and field measurements were conducted simultaneously; the village’s roads, squares, and green spaces were visited, and residents doing outdoor activities were randomly selected for the survey. The green spaces, squares, and streets were classified into eight categories:

A: space with fitness facilities;

B: unsheltered square;

C: spaces with shading from trees;

D: unshaded lawns;

E: green park spaces;

F: pavilions;

G: areas with shading from buildings;

H: roads

Subsequently, 450 local villagers (with ages ranging from 18 to 70; with a 1:1 ratio of men to women) were invited to participate in the survey. Finally, 412 valid questionnaires were obtained. Before the interviewees answered the questionnaire, they were asked to sit quietly for 20 min in a shaded area within a 1 min walk from the test site; the purpose of this was to achieve a uniform metabolic rate. The questionnaire results contain basic information about the interviewees and TSVs. Basic information includes personal characteristics, such as gender, age, weight, clothing, types of outdoor activities, and the time they spent outdoors. The TSV results include the residents’ perceptions and preferences of the meteorological parameters, overall thermal sensation, and comfort.

The evaluation of the residents’ thermal comfort characteristics is a comprehensive evaluation of an outdoor public space’s overall thermal environment. A five-point scale was used for the thermal-comfort evaluation (Table 2). The interviewees evaluated the overall comfort and thermal sensation characteristics of different types of spaces. The TSV corresponding to the temperature is presented with the ASHRAE55 seven-point scale [81] (Fig 3). The overall thermal comfort level is affected by many factors, such as the thermal environment, emotions, and physiology, which are related to the thermal sensation of the human body in the studied environment. The specific evaluation assignments are as follows:

**Fig 3.** **Thermal-sensation evaluation scales.**

**Table 2. Overall thermal comfort vote.**

| **Scale** | Very uncomfortable | Uncomfortable | Neutral | Slightly comfortable | Very comfortable |
| --- | --- | --- | --- | --- | --- |
| **TCV** | -2 | -1 | 0 | 1 | 2 |

(2) Thermal comfort indexes

The PET index was chosen based on the subjective data and field measurements to evaluate the outdoor thermal comfort characteristics of the village. The index comprehensively analyses the effects of all physiologically related weather indexes (the air temperature, relative humidity, wind speed, and mean radiation temperature) and personal parameters (the metabolic rate and clothing insulation level). The corresponding thermal sensation and thermal stress (physiological stress) are shown in Table 3 [82].

**Table 3. Classification of thermal sensation/stress on the PET scale.**

| **PET（℃）** | **＞41** | **[35，41）** | **[29，35）** | **[23，29）** | **[18，23）** | **[13，18）** | **[8，13）** | **[4，8）** | **＜4** |
| --- | --- | --- | --- | --- | --- | --- | --- | --- | --- |
| **Thermal Sensation** | Very hot | hot | Warm | Slightly warm | Neutral (Comfortable) | Slightly cool | Cool | Cold | Very cold |
| **Thermal Stress** | Extreme heat stress | Strong heat stress | Moderate heat stress | Slight heat stress | No thermal stress | Slight cold stress | Moderate cold stress | Strong cold stress | Extreme cold stress |

The RayMan software was applied to calculate the PET. It was assumed that the thermal resistance of clothing (0.9 clo) and active metabolic rate (80 W) remain constant during the calculation, and the physical parameters of the thermal environment were the input parameters (which included the temperature, humidity, wind speed, and mean radiation temperature (T_mrt_)). The relationship between the PET and mean TSV of the village’s thermal environment during the summer was studied, and the village’s neutral temperature range during the summer was determined.

$\text{MTSV=}\frac{\sum_{\text{n}}^{\text{j}} \text{TSV}}{\text{n}}$ （1）

where n is the number of TSVs of each thermal index, and MTSV represents the mean TSV.

### 2.4.2. Field measurements…

The objective study comprises a field study of the village to determine its microclimate. The relevant data were obtained with the help of photographs, observations, and measurements with scientific instruments. The ENVI-met model and measurement results were used to simulate the village’s microclimate. Finally, the ArcGIS 10.6 platform was applied for the analysis, and a thermal-comfort plan that is specific to the village was established.

(1) The selected microclimate indicators, such as the thermal environment parameters of different squares, the varying orientations of streets, and the locations of green spaces, were measured during the selected time period. Based on the measurement and analysis results of three different measurement points at a height of 1.5 m, the data/information of black globe temperature, air temperature, relative humidity, and wind speed at each measurement point were obtained. The specific measurement instruments are listed in Table 4.

**Table 4.** Experimental equipment used for the measurement of micrometeorological parameters.

| **Measured Parameter** | **Instrument** | **Range** | **Accuracy** |
| --- | --- | --- | --- |
| Air temperature | DT-83 | -20.0~60.0℃ | ±1℃（0℃~40℃）±2℃（-20℃~0℃&~40℃~60℃） |
| Relative humidity | DT-83 | 0.0~100.0% | ±3.5%RH（20%~80%）±5%RH（0~20%&80~100%） |
| Wind speed | GM8902 | 0~45m/s | ±3%±0.1 |
| Globe temperature | AZ8758 | 0~50℃ | ±3℃（15~40℃） |

(2) Mean radiant temperature

For outdoor measurements, the mean radiant temperature can be obtained by approximation from the black globe temperature, air temperature, and wind speed:

$T_{mrt}=[(Tg+273)^{4}+\frac{1.10*10^{8}Va^{0.6}}{\varepsilon D^{0.4}}(Tg-Ta)]^{\frac{1}{4}}-273$ （2）

where T_mrt_ is the mean radiant temperature; T_g_ is the black globe temperature (°C); T_a_ is the air temperature (°C); V_a_ is the wind speed (m/s); D is the globe diameter (0.05 m in this study), and Ɛ is the emissivity (0.95 for a black globe).

(3) Numerical simulation and thermal-comfort plan for the village

The model area of Maling Village is approximately 30 hm^2^. The June 14^th^, 2020 was chosen for the simulation. The simulation took 12 h (from 8:00), and the simulation intervals were 60 min. Based on the field investigation results, 91 satellite images, and ArcGIS 10.6, the study area was transformed into a base map with BMP format, which can be recognised by ENVI-Met 4.4.5. The map was imported into the sub-module ‘SPACE’ to establish the model. The maximal building height was set to 17.5 m, and the grid dimensions were set to 100 × 60 × 30. According to the dimensions and building height of the study area, the grid resolution was set to dx = dy = dz = 5 m, with four nested grids in the peripheral area. The initial microclimate data were the weather data of the macro climate environment on June 14^th^, 2020. The dominant wind direction was 170°; the model roughness length was 0.1, and the underlying surfaces of the model area were an asphalt road and a grey concrete road. The early simulation was based on the actual conditions of the measurement sites, and the later simulation was based on the village’s thermal-comfort plan. In addition, the pre-set scheme was used to simulate the microclimate, and the PET indexes were chosen to comprehensively evaluate the village’s thermal environment.

# 3. Results and Analysis

3.1. Subjective evaluation results

### 3.1.1. Time distribution of outdoor activities and preferences regarding thermal environment parameters

On working days during the summer, villagers prefer to perform outdoor activities in the morning and evening (mainly between 6:00 and 8:00 and after 16:00) because solar radiation is weaker during those times. The most active time period on weekends is between 18:00 and 20:00 (Table 5) because young people have more time for outdoor activities during evening hours. Therefore, the period during which residents go to public spaces and perform outdoor activities is advanced.

**Table 5. Main time periods for outdoor activities during the summer.**

| **Hour** | **Number of Workdays** | **Weekend Attendance** |
| --- | --- | --- |
| 6:00-8:00 | 65 | 28 |
| 8:00-10:00 | 45 | 57 |
| 10:00-12:00 | 30 | 38 |
| 12:00-14:00 | 10 | 17 |
| 14:00-16:00 | 33 | 30 |
| 16:00-18:00 | 75 | 87 |
| 18:00-20:00 | 88 | 95 |
| 20:00-21:00 | 66 | 60 |

According to the villagers’ preferences regarding the thermal environment parameters, the four meteorological parameters’ degrees of influence in relation to thermal comfort are as follows: air temperature > solar radiation > wind speed > and relative humidity (Fig. 4). 49% of the interviewees stated that the high summer heat had the greatest impact on the residents’ outdoor activities; 24% of the respondents believed that long-term exposure to the summer sun will cause discomfort. In addition, a few of the respondents reported that the summer wind cools the public spaces, improving thermal comfort characteristics. The majority of respondents reported that they do not think relative humidity significantly affects the thermal comfort level.

**Fig 4. Evaluation of the influence of certain characteristics on subjective comfort.**

### 3.1.2. Characteristics of thermal comfort and thermal sensation

(1) Evaluation results of overall thermal comfort

The summer survey results are shown in Fig 5. 67% of the respondents stated that the area shaded by trees has the highest comfort level, followed by the green spaces of parks (51%); 49% of the respondents stated that the thermal environment of unshaded lawns is ‘neutral’. In addition, 13% of the respondents stated that the road environment is the lowest in terms of comfort. The village’s public spaces in the order of high-to-low thermal-comfort are as follows: green spaces in parks > areas with shading from buildings > artificial structures > unsheltered squares > roads.

(A: space with fitness facilities; B: unsheltered square; C: shading from trees; D: unshaded lawns; E: green spaces in parks; F: pavilions; G: areas with shading from buildings; H: roads).

**Fig 5. Overall comfort votes for different spaces.**

(2) Overall evaluation results of TSV

The TSV distribution is shown in Fig 6; the values are mainly distributed in the moderate and over-hot ranges (TSV ≥ 0). According to the results, during the test period, the outdoor thermal environment of Maling Village is hot during the summer. The proportion of respondents who felt ‘TSV = 0’ was the highest; thus, most villagers have adapted well to the thermal environment. Most people reported feeling ‘hot’ and ‘very hot’ on the road and ‘hot’ in the unsheltered square. In addition, the respondents reported different thermal sensations for different types of spaces. This indicates that there are significant differences between different types of spaces in terms of in simultaneous thermal sensations.

(A: space with fitness facilities; B: unsheltered square; C: shading from trees; D: unshaded lawns; E: green spaces in parks; F: pavilions; G: areas with shading from buildings; H: roads).

**Fig 6. TSVs for different spaces.**

(3) Mean TSV and PET

The weighted mean values of the PET and TSV were calculated and fitted with a linear model. As shown in Fig 7, the overall scatter distribution is close to the fitting curve R^2^ = 0.8105. For a MTSV of 0–0.5, the neutral temperature of the human body corresponding to the PET is 23.77–27.23 ℃; the range of the neutral-temperature PET includes ‘no heat stress’ and ‘slight heat stress’. The results reveal a strong correlation between the PET and MTSV, and the degree of fit is good.

**Fig 7. Correlation between PET and MTSV.**

3.2 Objective evaluation results

### 3.2.1. Analysis of various thermal environments

(1) Measurement and analysis of the village square’s thermal environment.

As shown in Figs 8 and 9:

1. Mean air temperature: basketball square (Point 1) > fitness square (Point 14) > triangle square (Point 4) > pavilion square (Point 2).

2. In general, the relative humidity values of the pavilion square (Point 2) and triangle square (Point 4) are higher than those of Points 1 and 14; in addition, the relative humidity and air temperature are negatively correlated.

3. Mean wind speed: triangle square (Point 4) > pavilion square (Point 2) > basketball square (Point 1) > fitness square (Point 14). The wind speed of Point 4 is higher because Point 4 lies in a sunken square. The east side, which is near the road, is greatly affected by the natural wind direction; by contrast, the wind speed of Point 14 is the lowest because the wind is largely blocked by the building.

4. The black globe temperature at the four measurement points in the square begins to increase after 8:00. Therefore, its trend is consistent with the increase in solar radiation; the change range at Point 1 is the highest, indicating that it is most significantly affected by solar radiation. The black globe temperatures at Points 1 and 14 are high because the points are in an unsheltered square. Measurement Point 14 is only blocked by a few buildings, and Point 1 is open. Furthermore, the square is exposed to solar radiation for an extended period of time during the day. Therefore, the black globe temperatures at these two measurement points are high. The black globe temperature at Point 2 is the lowest and changes slightly; thus, the high buildings, which block the wind and provide shading, significantly influence the black globe temperature.

**Fig 8. Variation trends of microclimate parameters at each measurement point in the square.**

#### Fig 9. Mean variation of meteorological parameters at each measurement point in the square.

(2) Measurement and analysis of the village streets’ thermal environment.

As can be seen from Figs 10 and 11:

1. Mean air temperature: East–West Street of Market Road (Point 5) > Photovoltaic Road (Point 11) > North–South Street of Market Road (Point 3) > Park Road (Point 7) > Maling 5^th^ Group Road (Point 8) > West Street of primary school (Point 12) > North–South Street of School Road (Point 15). The maximal air temperature was measured at Point 5 at approximately 14:00 (38.7 °C); during this time, the air temperature at Point 15 was the lowest (34.9 °C; the difference between the maximum and minimum was 3.8 ℃). This was due to the building on the east side of Point 15 being higher than that on the west side and providing shading. According to the analysis results of the mean temperature at each measurement point on the East–West street, the mean air temperature at Point 12 is 32.6 ℃, which is lower than the temperatures at Points 5, 8, and 11. This difference in temperature is mainly due to the shading from the tall trees lining both sides of the road at Point 12; in contrast, Points 5, 8, and 11 have fewer trees in their vicinity.

2. The maximal black globe temperature of Point 5 was measured at approximately 14:00. (48.1 ℃). At this time, the black globe temperature was the lowest (40.9 ℃) at Point 15. Hence, there is a difference of 7.2 ℃ between the maximal and minimal temperatures. In addition, the black globe temperature curve of Points 12 and 15 is relatively stable, and the mean black globe temperature is lower than at other points. The results show that lining a given street with trees and buildings can effectively reduce the impact of solar radiation

3. The hourly variation curve of the wind speed showed no evident trend during the entire measurement process due to the significant, random variations in wind direction. However, when the building layouts are relatively consistent, the fluctuation in wind speed is relatively low for a street lined with trees. Therefore, plants above a certain height significantly reduce the wind speed and improve thermal comfort for a village street.

4. Overall, the mean relative humidity is highest at Point 15, followed by Point 12. Since there are buildings and trees that provide shading for Points 15 and 12, respectively, the mean relative humidity is relatively high. By contrast, the unsheltered streets are greatly affected by solar radiation during the day; this leads to a low mean relative humidity of the surrounding air.

**Fig 10. Variation trend of microclimate parameters at each measurement point in the street.**

#### Fig 11. Mean variation of meteorological parameters at each measurement point in the street.

(3) Measurement and analysis of the village green spaces’ thermal environments.

As shown in Figs 12 and 13:

1. The order of the mean air temperature is as follows: green space on the south side of the village (Point 13) > green space on the west side of the village (Point 6) > green space on the west side of Park Road (Point 10) > green space on the east side of Park Road (Point 9). The air temperature increases continuously after 8:00 and reaches its maximum at 14:00. The air temperature at Point 13 reaches 36.8 ℃, and the air temperature at Point 9 is the lowest (31.9 ℃, which corresponds to a difference of 4.9 ℃).

2. The air temperature values at Points 9 and 10 gradually increased from 10:00 to 14:00. The change curve is relatively stable because the village employed special personnel responsible for watering and maintaining the plants in the park during this period. The ejected water lowers the air temperature. This effect is similar to direct evaporative cooling. Therefore, during this period, the air temperature of the park was lower than those of the other two measurement points; the maximal difference is approximately 2.7 ℃. Hence, human intervention has a short-term and rapid effect on humidity. The change trend of the relative humidity of the green space in the village is inversely proportional to the air temperature: the higher the air temperature, the lower the relative humidity. For example, there are many tree species at Points 9 and 10; their transpiration can increase the relative humidity of the surrounding air. In addition, the air humidity at each green-space measurement point were higher in the mornings and evenings.

3. The highest black globe temperature is measured at Point 13 at 14:00 (46.1 ℃). The corresponding black globe temperatures of the two green spaces in the park are the lowest (below 40 ℃). This is mainly due to the fact that the plants at Point 13 are wild and of the low ground-cover type; the area is primarily covered by unsheltered grassland without planted trees.

4. Points 9 and 10 are surrounded by trees and buildings. Therefore, the wind speeds are lower and more stable at these two points than at the other two measurements points.

**Fig 12. Variation trends of microclimate parameters at each measurement point in green space.**

**Fig 13. Mean variation of meteorological parameters at each measurement point of green space.**

### 3.2.2 Comparison of thermal environments

The mean values of three different space climate impact parameters were studied to compare the corresponding thermal environments; Table 6 presents the results.

**Table 6. Mean values of the microclimate parameters of different types of spaces.**

| **Space Type** | **Mean Air Temperature (℃)** | **Mean Relative Humidity (%)** | **Mean Black Globe Temperature (℃)** | **Mean Wind Speed(m/s)** |
| --- | --- | --- | --- | --- |
| Village square | 34.06 | 61.89 | 36.81 | 1.21 |
| Village street | 34.12 | 55.08 | 38.78 | 1.01 |
| Village green space | 30.57 | 72.93 | 34.20 | 1.19 |

The following conclusions can be drawn:

(1) The temperature of the green space of the village is lower than the temperatures of the roads and squares; the highest difference is 3.55 °C. This difference is due to the shade, which can effectively reduce the influence of solar radiation, and the transpiration of plants, which absorb the surrounding heat. The watering of plants also helps to cool the area.

(2) The mean relative humidity values of the squares and roads are lower than those of the green spaces; more specifically, the relative humidity of the road is the lowest (approximately 17.85% lower than that of the green space of the village). The village road and square have hard underlying surfaces; most of the roads are made of asphalt pavement, whereas some have been constructed with cement pavement; the square is mainly constructed of white ceramic tile and red square bricks, while the underlying surface of the green space is primarily grassland. The ground of the road is exposed to sunlight for an extended period of time and consists of asphalt and cement pavement; this causes the road to heat up quickly, which reduces the relative humidity in the air. Nevertheless, the evaporation of water and the transpiration of plants increase the air humidity in the village park, significantly increasing the relative humidity.

(3) According to Table 7, the black globe temperature of the village road is (approximately 4.58 ℃) higher than that of the green space. With the exception of temperature values at Points 12 and 15 on the village roads, the black globe temperature values of the other roads are high due to the lack of street trees and shading from buildings on both sides. In addition, the village has a large green area with dense trees, which effectively block the sunlight and reduce the mean black globe temperature. Consequently, the mean black globe temperature of the village’s green space is much lower than that of the road.

(4) The mean wind speeds of each space type exhibited little deviations without evident regularity. In addition, the wind speed at the square exceeded the mean wind speed. As most measurement points are located at an intersection or on the roadside, the wind speed cannot be significantly reduced by trees or buildings.

### 3.2.3 Simulation of factors that affect thermal comfort

The dominant wind direction of the village was south–east on June 14^th^ ,2020. The wind speed distribution within the study area is shown in Fig. 14(a); the wind speed was generally 0.00–2.45 m/s. For some open public spaces in the middle of the study area, the wind speed reached 0.54–1.36 m/s. Due to canyon effect, the highest wind speed reach between 1.36 and 2.45 m/s at the east side of the village, where buildings are tall. Moreover, the wind speeds at the south and north of the village are greater (1.36–1.63 m/s); this is primarily because the areas are not blocked by buildings. The terrain on the south side is high; the site is spacious; and the overall space is well ventilated. In addition, the solar radiation intensity around the village buildings remains in a comfortable range (indicated by the blue colour). However, owing to the village’s low buildings and the relatively sparse plants, the solar radiation intensities of the village road and square are high at 14:00.

The simulation map of the air temperature and relative humidity in the village is shown in Figs 14 (c) and (d). The temperature of the study area increases gradually from 30.2 to 38.2 ℃ (from dark blue to red). Owing to the tall buildings in the east of the village and the relatively open layout, the temperature is the lowest in this area. This value is followed by that of the green area in the middle of the village (green and light-yellow zones in the map). This area includes a lot of green space, which has an evident cooling effect at 14:00. In addition, the temperature values of the north and west sides of the village are the highest, whereas the temperature values in the east and south of the denser areas are lower. Therefore, shading provided by buildings and trees can reduce the ambient temperature. The overall humidity of the village was found to range from 35.52% to 80.32%. According to the current conditions, the east side of the village has much open space, rich vegetation, and high air mobility, all of which lead to a high relative humidity. In contrast, due to the high building density on the streets of Market Road, the hard pavement of the public space, and poor vegetation, the relative humidity is the lowest.

**Fig 14. Simulation results of the thermal environment.**

### 3.2.4 Thermal-comfort plan for the village

From the site measurements, microclimate simulations, and current land use map of the village (Fig 3), it can be concluded that the factors affecting thermal comfort are the geographical location, nature of the underlying surface, plant configuration, types of outdoor activities, and building layout, among others. The aim of a thermal-comfort plan is to mitigate the deficiencies of public spaces. The thermal comfort level and planning approaches for the outdoor space of the village are listed in Table 7.

**Table 7. Thermal comfort level and planning approaches for outdoor public space in villages**

| **Number** | **Space Type** | **Specific Geographical Location** | **Underlying Surface Properties** | **Plant Configuration** | **Outdoor Activity Condition** | **Solutions** |
| --- | --- | --- | --- | --- | --- | --- |
| 1 | Square | Close to highway | Concrete ground | No | The flow of people is maximal at night. | Replace original hard concrete with permeable paving bricks to ensure rainwater infiltration and self-regulation of thermal environment. |
| 2 | Square | Intersection of West–East Street of Market Road and highway | White tile | No | Low flow of people; residents mainly stay for a short time; no fitness facilities. | Replace paving materials, reduce area with bricks and increase green space. |
| 3 | Road | Middle section of North–South Street of Market Road | Asphalt pavement | The road has plants one sides. | Main roads with high flow of people, primarily because of traffic; there is typically no extended stoppage. | Enlarge green areas next to roads, match plants reasonably and replace tree species with fewer leaves with native plants with higher leaf density index values. |
| 4 | Square | Intersection of North–South Street, Market Road, and highway | Red square bricks | Only a few vines cling to the wooden gallery frame. Solar radiation can still enter the lower space through some green plants during the summer. | Flow of people is concentrated at night; area has fitness facilities. | Increase the square green area, while ensuring ventilation and lighting; in addition, increase the number of shading trees. |
| 5 | Road | Middle section of West–East Street of Market Road | Asphalt pavement | On both sides of the road, the bifurcation point of the street trees is low, and the leaf density is low; the low heights of the plants does not provide sufficient shade. | Main roads with high flow of people and traffic, usually without extended stoppage. | Increase the roadside trees on both sides of the road; replace the trees with low branching points with those with high branching points, and replace trees with low leaf density index values with native plants with higher leaf density index values to enhance the ventilation performance and provide shading on the road during the summer. |
| 6 | Green space (irregular shape) | West side of East–West Street of Market Road | Grassland | Many wild plants block the ventilation space. | Low pedestrian flow; people generally do not stop here. | Increase manual maintenance; enrich plant levels, and improve the openness of space. |
| 7 | Road | North side of Park Road | Asphalt pavement | More shrub species than arbour species on both sides of the road. | Secondary trunk roads with low pedestrian flow; people generally do not stop here. | Plant trees with dense leaves. |
| 8 | Road | Middle section of Maling 5^th^ Group Road | Asphalt pavement | North side of road has low-growing shrubs, and south side has street trees; nevertheless, overall plant spacing is great, and trees are small. | Main access road; the stoppage time is short. | Increase trees on south side of road; reduce tree spacing in some areas to an appropriate distance and replace original tree species with those with high leaf density index values. |
| 9 | Green space (rectangle) | East side of Park Road | Grassland | Middle part of green space constitutes a microtopography with 0.5 m height; plant community with rich natural landscapes and densely planted trees, shrubs, and grass (e.g. Koelreuteria paniculata, Ginkgo biloba, Sophora japonica, Rosa chinensis). | In the morning, special personnel water and maintain the plants in the park. There is a large flow of people in the morning and afternoon, and the main activities are chatting, watching, and walking. | Adjust plant density and some varieties in the middle micro-terrain area, increase ventilation performance of park space and replace road surface material of garden road with permeable pavement. |
| 10 | Green space (polygon) | West side of Park Road | Grassland | Plant species are abundant and mainly densely planted; however, there are more plants with low branch points (e.g. Pinus bungeana, cedar, tower pine, Sabina chinensis, and Prunus cerasifera Ehrh.). | There is a large flow of people in the afternoon. In the morning, special personnel water and maintain the plants in the park. The main activities of the villagers are chatting, watching and walking. | Reduce planting of plants with low branch points and increase ventilation performance of park space. Tree species with dense leaves and high branching points such as Magnolia, purple leaf plum, acacia, sweet-scented Osmanthus, and tree heather can be added; in addition, trees with a better landscape effect such as ginkgo and cherry blossoms can be planted to enrich the landscape level and increase ventilation. |
| 11 | Road | Photovoltaic Road | Asphalt pavement | Distributed on one side of the road. | Secondary trunk roads with low pedestrian flow; people generally do not stop here. | Plant more street trees on both sides of road for more tree shading. |
| 12 | Road | West Street of Primary School | Concrete ground | Walnut and other trees are planted on north side of road, buildings are located on south side, and some illegal buildings block the ventilation effect on the south side of the village. | Secondary trunk road with low flow of people. | Keep original walnut trees on the road, demolish illegal buildings on the south side of the street in order to increase the permeability. |
| 13 | Green space (ribbon) | North side of fishpond | Grassland | Lawn is sparse and unsheltered; area is dominated by ground-cover plants, without trees and shrubs. | Flow of people is maximal in the afternoon. | Plant tree species with high branches (good ventilation), provide space below the tree crowns, and ensure shading while reducing the barrier of the lower layer to the south wind. |
| 14 | Square | Intersection of Photovoltaic Road and North–South Street of School Road | Red square bricks | Plant coverage rate is low, only some low arbours. | High flow of people; there are fitness facilities in the open-air square, and the villagers perform outdoor activities in the morning, noon, and evening; at night, there are more young people. The main activities are morning exercises, parent–child entertainment, and fitness. | Plant deciduous tree species with high leaf density indexes in order to get sunshade in summer and more sunshine in winter. Different changes of four levels of plants, such as Ginkgo biloba, Koelreuteria, Fraxinus chinensis, and Sophora japonica; construct temporary sunshade facilities, such as sunshade umbrellas, to improve the overall landscape and thermal environment of the square. |
| 15 | Road | North–South Street of School Road | Asphalt pavement | Distributed on one side of the road, and west side of the road has low trees and shrubs. | Main roads have a high flow of people, which changes during the day. | Increase the height of some buildings on the east side and replace the trees on the west side of the original road with trees with higher leaf densities. |

Based on the previously presented analysis, the thermal-comfort plan was classified into four parts: building layout optimisation, square layout optimisation, road layout optimisation, and green space layout optimisation. The sunshade mode of the public space in the village combines shading from buildings and plants. In addition, the plan includes three new squares (S5, S6, and S7) and two new green spaces (G5 and G6), and the layout of the local roads is improved. The present thermal-comfort map and proposed design plan are shown in Figs 15 (a) and (b). The details are as follows:

(1) Optimisation of building layout: the building layout can affect the microclimate of the outdoor space and control the wind direction to a certain extent. According to previous studies [77, 83-85], the spatial structure is mainly controlled by the architectural layout. The spacing, orientation, floor height, and shape of a building will also affect the air flow. The aim of this study was to determine a better architectural layout while maintaining the overall architectural layout. The terrain on the south side of the village is high and is currently used as woodland. During the summer, the air flows from the forest to the open area of the village (as such, it is called ‘forest wind’). There are two ways to improve the thermal environment. First, the illegal buildings on the south side of the village should be demolished to improve ventilation. Second, the village land should be designed according to the ventilation needs and intensively. Specifically, the buildings on the south side should be reduced in number by transforming the area into a green space. The fewer buildings on the south side can be compensated by increasing the floor height of the buildings on the north side, reducing the local solar radiation intensity.

(2) Optimisation of square layout: the thermal environment of the square is mainly optimised by improving shading, changing the nature of the underlying surface, and increasing spatial openness. More specifically, thermal comfort can be improved ① by adding more green space to the square, creating shaded areas with plants, reducing the direct exposure of villagers to solar radiation in summer, and improving the space utilisation of the square. Furthermore, three squares can be added (S5, S6, S7): S5 is a waterscape square with a shallow water pool in the middle. The waterscape square is surrounded by deciduous trees; the site is equipped with tension film and sunshade. The square S6 is a tree square, and S7 is a basketball hall square. In addition, the S4 square area can be optimised. ② The underlying surface should be replaced to reduce the coverage of the hardened ground of the square, thus reducing the heat stress. In addition to installing permeable tiles in the S1 and S2 squares, the new parking lot in the S7 area is built with grass-planting bricks, which increase the green area and enable the infiltration of rainwater so that sufficient parking space and a cool and comfortable environment are provided for outdoor activities on hot days.

(3) Optimisation of road layout: the comfort of the road space is improved by improving the connectivity of the traffic corridor and increasing the green space next to the road. For example, the road layouts on the left side of R2 and R4 can be changed, and the original layout of the road can be replaced with the north–south layout for better road ventilation. In addition, the road on the east side of the North–South Road of the school can be extended such that the space can be fully exploited. Moreover, street trees can be planted on both sides of the street; the selected deciduous umbrella-shaped trees increase the shading level and create a cool space during the summer.

(4) Optimisation of green space: the green space can be optimised by increasing the green space, reasonably collocating the plant levels, and selecting suitable plant varieties. More specifically, ① the green space should be increased, and broad-leaved trees should be planted. Owing to the good shading effect and leaf transpiration, the temperature under and around broad-leaved trees is relatively low, which improves the thermal environment. Furthermore, the original green space (G3) can be expanded, new residential green space (G5) can be added, and a small garden (G6) can be integrated into the village map. ② the different requirements for thermal comfort during the winter and summer should be considered. The proposed plan includes deciduous plants, evergreen plants, and local trees with high branching points. For example, deciduous trees with broad canopies can be planted around the garden road in the green space, and trees, shrubs, and grass land should be distributed to properly provide adequately shaded areas in summer and sufficient radiation in winter.

(a)

(b)

**Fig 15. (a) Current map of the village’s public spaces. (b) Thermal-comfort plan of the village’s public spaces.**

(5) To evaluate the proposed plan, PET indicators were used to simulate the thermal environment of the village (Fig 16). According to the comprehensive analysis results of its thermal environment, the neutral temperature of the outdoor public space is 23.77–27.23 ℃ at 14:00 during the summer. The areas reported to be ‘very hot’ sensation were outside the boundaries of the village’s public spaces. The areas reported to be ‘hot’ were the main roads, and the areas reported to be ‘warm’ were the green spaces and squares. Due to the large green area and many tall buildings on the east side, the PET remains low. Therefore, a plan for the thermal environment of the village can improve the overall thermal environment, facilitating the cooling effect on village roads and squares in particular.

**Fig 16. Spatial distribution of PET in the village.**

# 4. Discussion

(1) Analysis of differences before and after realising the thermal environment plan. Researchers that have studied the thermal environments of urban environments, such as urban parks [72], squares [74], and streets [57], have focused on the measurement and evaluation of indicators. Specific thermal-comfort plans have not been investigated until now. In this study, the influencing factors of the village’s thermal environment were studied by evaluating the village’s thermal environment and conducting ENVI-met simulations. The resulting, feasible thermal-comfort scheme for the village is presented in Fig 15 (a). The village’s current thermal environment was studied with GIS spatial interpolation and ENVI-met simulations (Fig 17). The results of the two methods were also compared. The GIS spatial interpolation method reflects the evident variation characteristics of the microclimate parameters at each measurement point. Nevertheless, the method has limitations; some high value points may be neglected in the analysis; for example, Fig 17 (a) presents an area with a lower temperature between Points 6 and 11; by contrast, the simulation results (Fig 14 (c)) shows an area with a higher temperature between Points 6 and 11. Thus, the software provides more comprehensive results. Moreover, the building height has an evident influence on the wind speed, temperature, solar radiation, and humidity in the village. Nevertheless, the building height must meet the thermal comfort and overall stylistic and cultural requirements of the village. The shortcomings of the spatial analysis method can be overcome by performing additional ENVI-met simulations.

The data from the subjective questionnaire survey and agree well with the objective analysis and reveal some evident problems. For example, the roads and squares have poor thermal comfort conditions, which cause a ‘hot’ sensation during the summer. Therefore, in the future, more attention should be paid to the thermal comfort conditions of the streets and squares in Maling Village. During the summer, the villagers perform outdoor activities more frequently in cooler areas. A comfortable temperature, humidity, and wind environment can effectively reduce the heat and prolong the residents’ outdoor activities. The layout should consider the shapes of the open spaces, plant configurations, nature of the underlying surface, and building heights and layouts as well as other factors.

**Fig 17. Spatial distribution map of the thermal environment at each measurement point.**

(2) To improve the overall thermal comfort level in the village, a green, ecological, and adequate ‘matrix–corridor patch’ model should be created based on the village resources. Maling Village, which has good ecological resources, is surrounded by hills with lush forests and grass in the south. The proposed thermal-comfort plan for the village (e.g. the matrix utilisation, corridor construction, and patch optimisation) should exploit the resources and advantages of the village, .

# 5. Conclusion

(1) Spatiotemporal distribution of the village’s thermal environment. In general, the overall thermal comfort conditions were the worst in the village streets, and thus villagers are less willing to stay in these areas for extended periods of times. The green spaces were frequently visited in the morning and afternoon, while the squares were more often visited after supper. The mean air temperature of the East–West Street is higher than that of the North–South Street, the temperature of the green space in the middle of the village is lower than that at the border of the village, and the temperature of the square on the south side of the village is higher than that on the north side. Furthermore, the temperature of the public space increases to its maximum value until 14:00 and remains at that value from 14:00 to 16:00.

(2) Factors that influence the thermal comfort in public spaces of the village. During the summer, the effects of the temperature, solar radiation, wind speed, and relative humidity on the subjective comfort of the village’s outdoor space gradually decrease. The factors that influence the thermal comfort level of public spaces are as follows: shape of the public space > plant configuration > building height and layout > nature of underlying surface > street directions > nature of outdoor activity.

(3) Thermal-comfort plan for the village’s public spaces. The thermal-comfort plan in Fig 18 is based on field measurements, simulations, and literature reviews.

**Fig 18. Thermal-comfort plan for the village’s public spaces.**

In future studies, the thermal comfort level of public spaces should be considered in the construction layout of the village. A higher thermal comfort level creates a healthier environment, which motivates villagers to perform more outdoor activities.

# Acknowledgments

First of all. I would like to thank the support of the project funding. I am very grateful to my tutor for helping me to complete the topic selection and conception of the thesis, and gave me a lot of valuable suggestions on the revision of my thesis, so that my paper has been constantly improved. In addition, in the process of completing my thesis, I would like to express my heartfelt thanks to my family and friends who care and help me.

# Author Contributions

All authors contributed to the paper. Q.F. contributed to the conception of the study and as the main writer of the manuscript; F.D. performed the site investigation and data analyses; F.D. wrote the manuscript with the supervision from Q.F., and F.D. acted as a corresponding author and the second author. Q.F. reviewed this research. H.L. and C.Z. provided suggestions on local situation and acted as the third author. All authors have read and agreed to the published version of the manuscript.

**Competing interests:** The authors have declared that no competing interests exist.

# References

[1]	United Nations World Urbanization Prospects .2018. [Available from: <https://esa.un.org/unpd/wup/Download/>. .

[2]	Wen B, Burley JB. Expert Opinion Dimensions of Rural Landscape Quality in Xiangxi, Hunan, China: Principal Component Analysis and Factor Analysis. Sustainability. 2020;12(4):18.

[3]	Zou L, Liu Y, Yang J, Yang S, Hu X. Quantitative identification and spatial analysis of land use ecological-production-living functions in rural areas on China's southeast coast. Habitat International. 2020;100:102182.

[4]	Huang QD, Xu JJ, Qin H, Gao XY. Understanding Land Use and Rural Development in the National Scheme of Village Relocation and Urbanization in China: A Case Study of Two Villages in Jiangsu Province. Sustainability. 2018;10(9):13.

[5]	Liu J, Wang L, Yoshino Y, Liu Y. The thermal mechanism of warm in winter and cool in summer in China traditional vernacular dwellings. Building & Environment. 2011;46(8):1709-15.

[6]	Xiao, Zhao, Sun, Guo, Sang. Sustainability Dynamics of Traditional Villages: A Case Study in Qiannan Prefecture, Guizhou, China. Sustainability. 2019;12(1):314.

[7]	Gehl J, Gemzøe L. Public spaces, public life. How to Study Public Life. 2004;2(1).

[8]	Ma K, Tang X, Ren Y, Wang Y. Research on the Spatial Pattern Characteristics of the Taihu Lake "Dock Village" Based on Microclimate: A Case Study of Tangli Village. Sustainability. 2019;11(2).

[9]	Tang L, Zhang N. A study on the thermal environment of historical villages with 'comb-like' layout in southern China. Indoor and Built Environment. 2018;27(5):658-64.

[10]	Kántor N, Chen L, Gál CV. Human-biometeorological significance of shading in urban public spaces—Summertime measurements in Pécs, Hungary. Landscape & Urban Planning. 2018;170:241-55.

[11]	Gold E. The effect of wind, temperature, humidity and sunshine on the loss of heat of a body at temperature 98°F. Quarterly Journal of the Royal Meteorological Society. 2010;61(261):316-46.

[12]	Zhang LLL, Wei D, Hou YY, Du JF, Liu Z, Zhang GM, et al. Outdoor Thermal Comfort of Urban Park-A Case Study. Sustainability. 2020;12(5):23.

[13]	Chen L, Wen Y, Zhang L, Xiang WN. Studies of thermal comfort and space use in an urban park square in cool and cold seasons in Shanghai. Building and Environment. 2015;94(DEC.PT.2):644-53.

[14]	Vanos JK, Warland JS, Gillespie TJ, Kenny NA. Review of the physiology of human thermal comfort while exercising in urban landscapes and implications for bioclimatic design. International Journal of Biometeorology. 2010;54(4):319-34.

[15]	Knez I, Thorsson S. Thermal, emotional and perceptual evaluations of a park: Cross-cultural and environmental attitude comparisons. Building & Environment. 2008;43(9):1483-90.

[16]	Andrade H, Alcoforado MJ, Oliveira S. Perception of temperature and wind by users of public outdoor spaces: relationships with weather parameters and personal characteristics. International Journal Of Biometeorology. 2011;55(5):665-80.

[17]	Xi TY, Li Q, Mochida A, Meng QL. Study on the outdoor thermal environment and thermal comfort around campus clusters in subtropical urban areas. Building And Environment. 2012;52:162-70.

[18]	Lai D, Guo D, Hou Y, Lin C, Chen Q. Studies of outdoor thermal comfort in northern China. Building & Environment. 2014;77:110-8.

[19]	Qi Y, Liu J, Lai D, Zhang H, Yoshino H. Large-Scale and Long-Term Monitoring of the Thermal Environments and Adaptive Behaviors in Chinese Urban Residential Buildings. Building and Environment. 2020;168:106524.

[20]	Zaki, Toh, Yakub, Saudi M, Muhammad-Sukki. Effects of Roadside Trees and Road Orientation on Thermal Environment in a Tropical City. Sustainability. 2020;12(3):1053.

[21]	He X, An L, Hong B, Huang B, Cui X. Cross-cultural differences in thermal comfort in campus open spaces: A longitudinal field survey in China's cold region. Building and Environment. 2020;172:106739.

[22]	Liu Z, Jin Y, Jin H. The Effects of Different Space Forms in Residential Areas on Outdoor Thermal Comfort in Severe Cold Regions of China. International Journal of Environmental Research and Public Health. 2019;16(20):3960.

[23]	Niu J, Liu J, Lee T-c, Lin ZJ, Mak C, Tse K-T, et al. A new method to assess spatial variations of outdoor thermal comfort: Onsite monitoring results and implications for precinct planning. Building and Environment. 2015;91:263-70.

[24]	Morakinyo TE, Lam YF. Simulation study on the impact of tree-configuration, planting pattern and wind condition on street-canyon's micro-climate and thermal comfort. Building and Environment. 2016;103:262-75.

[25]	Morakinyo TE, Lau KK-L, Ren C, Ng E. Performance of Hong Kong's common trees species for outdoor temperature regulation, thermal comfort and energy saving. Building and Environment. 2018;137:157-70.

[26]	Sun S, Xu X, Lao Z, Liu W, Li Z, García EH, et al. Evaluating the impact of urban green space and landscape design parameters on thermal comfort in hot summer by numerical simulation. Building and Environment. 2017;123:277-88.

[27]	Limona SS, Al-hagla KS, El-sayad ZT. Using simulation methods to investigate the impact of urban form on human comfort. Case study: Coast of Baltim, North Coast, Egypt. Alexandria Engineering Journal. 2019;58(1):273-82.

[28]	Lu J, Li QS, Zeng LY, Chen J, Liu GX, Li YC, et al. A micro-climatic study on cooling effect of an urban park in a hot and humid climate. Sustainable Cities And Society. 2017;32:513-22.

[29]	Ma X, Fukuda H, Zhou D, Gao WJ, Wang MY. The study on outdoor pedestrian thermal comfort in blocks: A case study of the Dao He Old Block in hot-summer and cold-winter area of southern China. Solar Energy. 2019;179:210-25.

[30]	Ehsan S, John B. Passive activity observation (PAO) method to estimate outdoor thermal adaptation in public space: case studies in Australian cities. International Journal of Biometeorology. 2018:1-12.

[31]	Johansson E, Yahia MW, Arroyo I, Bengs C. Outdoor thermal comfort in public space in warm-humid Guayaquil, Ecuador. International Journal of Biometeorology. 2017;62(4).

[32]	da Silveira Hirashima SQ, Katzschner A, Ferreira DG, de Assis ES, Katzschner L. Thermal comfort comparison and evaluation in different climates. Urban Climate. 2018;23:219-30.

[33]	Ali SB, Patnaik S. Thermal comfort in urban open spaces: Objective assessment and subjective perception study in tropical city of Bhopal, India. Urban climate. 2018;24:954-67.

[34]	Zhang A, Bokel R, van den Dobbelsteen A, Sun Y, Huang Q, Zhang Q. An integrated school and schoolyard design method for summer thermal comfort and energy efficiency in Northern China. Building & Environment. 2017;124(nov.):369-87.

[35]	Zlch T, Rahman MA, Pfleiderer E, Wagner G, Pauleit S. Designing public squares with green infrastructure to optimize human thermal comfort. Building and Environment. 2018;149.

[36]	Nikolopoulou M, Baker N, Steemers K. Thermal comfort in outdoor urban spaces: Understanding the Human parameter. Solar Energy. 2001;70(3):227-35.

[37]	Nikolopoulou M, Lykoudis S. Thermal comfort in outdoor urban spaces: Analysis across different European countries. Building & Environment. 2006;41(11):1455-70.

[38]	Morakinyo TE, Kong L, Lau KL, Yuan C, Ng E. A study on the impact of shadow-cast and tree species on in-canyon and neighborhood's thermal comfort. Building & Environment. 2017;115(APR.):1-17.

[39]	Morakinyo TE, Lau KL, Ren C, Ng E. Performance of Hong Kong's common trees species for outdoor temperature regulation, thermal comfort and energy saving. Building and Environment. 2018:157-70.

[40]	Bruse M, Fleer H. Simulating surface–plant–air interactions inside urban environments with a three dimensional numerical model. Environmental Modelling & Software. 1998;13(3-4):373-84.

[41]	Rui LY, Buccolieri R, Gao Z, Ding WW, Shen JL. The Impact of Green Space Layouts on Microclimate and Air Quality in Residential Districts of Nanjing, China. Forests. 2018;9(4):20.

[42]	Fahmy M, Sharpies S. On the development of an urban passive thermal comfort system in Cairo, Egypt. Building & Environment. 2009;44(9):1907-16.

[43]	Yang X, Zhao L, Bruse M, Meng Q. Evaluation of a microclimate model for predicting the thermal behavior of different ground surfaces. Building & Environment. 2013;60(FEB.):93-104.

[44]	Hofman J, Samson R. Biomagnetic monitoring as a validation tool for local air quality models: A case study for an urban street canyon. Environment International. 2014;70(sep.):50-61.

[45]	Kruger EL, Minella FO, Rasia F. Impact of urban geometry on outdoor thermal comfort and air quality from field measurements in Curitiba, Brazil. Building and Environment. 2011;46(3):621-34.

[46]	Wania A, Bruse M, Blond N, Weber C. Analysing the influence of different street vegetation on traffic-induced particle dispersion using microscale simulations. Journal of Environmental Management. 2012;94(1):91-101.

[47]	Morakinyo TE, Lam YF. Simulation study of dispersion and removal of particulate matter from traffic by road-side vegetation barrier. Environmental Science & Pollution Research. 2015:1-14.

[48]	Zhang L, Zhan Q, Lan Y. Effects of the tree distribution and species on outdoor environment conditions in a hot summer and cold winter zone: A case study in Wuhan residential quarters. Building and Environment. 2018;130:27-39.

[49]	Morakinyo, TE, Lau, KKL, Ren. Performance of Hong Kong's common trees species for outdoor temperature regulation, thermal comfort and energy saving. BUILD ENVIRON. 2018;2018,137(-):157-70.

[50]	Milosevic DD, Bajsanski IV, Savic SM. Influence of changing trees locations on thermal comfort on street parking lot and footways. Urban Forestry & Urban Greening. 2017;23:113-24.

[51]	Thorsson S, Lindqvist M, Lindqvist S. Thermal bioclimatic conditions and patterns of behaviour in an urban park in Göteborg, Sweden. International Journal of Biometeorology. 2004;48(3):149-56.

[52]	Taleghani M, Kleerekoper L, Tenpierik M, Dobbelsteen AVD. Outdoor thermal comfort within five different urban forms in the Netherlands. Building & Environment. 2015;83(jan.):65-78.

[53]	Aboelata A. Vegetation in different street orientations of aspect ratio (H/W 1:1) to mitigate UHI and reduce buildings' energy in arid climate. Building And Environment. 2020;172:16.

[54]	Hamdan DMA, De Oliveira FL. The impact of urban design elements on microclimate in hot arid climatic conditions: Al Ain City, UAE. Energy and Buildings. 2019;200:86-103.

[55]	Acero JA, Koh EJK, Pignatta G, Norford LK. Clustering weather types for urban outdoor thermal comfort evaluation in a tropical area. Theoretical And Applied Climatology. 2020;139(1-2):659-75.

[56]	Acero JA, Koh EJY, Li XX, Ruefenacht LA, Pignatta G, Norford LK. Thermal impact of the orientation and height of vertical greenery on pedestrians in a tropical area. Building Simulation. 2019;12(6):973-84.

[57]	Ali-Toudert F, Mayer H. Effects of asymmetry, galleries, overhanging faades and vegetation on thermal comfort in urban street canyons. Solar Energy. 2007;81(6):742-54.

[58]	Lu J, Li Q, Zeng L, Chen J, Liu G, Li Y, et al. A micro-climatic study on cooling effect of an urban park in a hot and humid climate. Sustainable Cities and Society. 2017:513-22.

[59]	Chow WTL, Brazel AJ. Assessing xeriscaping as a sustainable heat island mitigation approach for a desert city. Building and Environment. 2012;47(Jan.):170–81.

[60]	Fang Z, Feng X, Liu J, Lin Z, Mak CM, Niu J, et al. Investigation into the differences among several outdoor thermal comfort indices against field survey in subtropics. Sustainable cities and society. 2019;44:676-90.

[61]	Fanger, P. O. Thermal comfort: analysis and applications in environmental engineering. Thermal Comfort Analysis & Applications in Environmental Engineering. 1972.

[62]	Gao S, Li Y, Wang YA, Meng XZ, Zhang LY, Yang C, et al. A human thermal balance based evaluation of thermal comfort subject to radiant cooling system and sedentary status. Applied Thermal Engineering. 2017;122:461-72.

[63]	Blazejczyk K, Epstein Y, Jendritzky G, Staiger H, Tinz B. Comparison of UTCI to selected thermal indices. International Journal of Biometeorology. 2012;56(3):515-35.

[64]	Park S, Tuller SE, Jo M. Application of Universal Thermal Climate Index (UTCI) for microclimatic analysis in urban thermal environments. Landscape & Urban Planning. 2014;125:146-55.

[65]	Lin TP, Tsai KT, Liao CC, Huang YC. Effects of thermal comfort and adaptation on park attendance regarding different shading levels and activity types. Building & Environment. 2013;59(JAN.):599-611.

[66]	Xu X, Sun S, Liu W, Garcia EH, He L, Cai Q, et al. The cooling and energy saving effect of landscape design parameters of urban park in summer: A case of Beijing, China. Energy and Buildings. 2017;149(aug.):91-100.

[67]	Lin TP, Tsai KT, Hwang RL, Matzarakis A. Quantification of the effect of thermal indices and sky view factor on park attendance. Landscape and Urban Planning. 2012;107(2):137-46.

[68]	Niu JL, Liu JL, Lee TC, Lin Z, Mak C, Tse KT, et al. A new method to assess spatial variations of outdoor thermal comfort: Onsite monitoring results and implications for precinct planning. Building And Environment. 2015;91:263-70.

[69]	Mayer H, Holst J, Dostal P, Imbery F, Schindler D. Human thermal comfort in summer within an urban street canyon in Central Europe. Meteorologische Zeitschrift. 2008;17(3):241-50.

[70]	Amindeldar S, Heidari S, Khalili M. The effect of personal and microclimatic variables on outdoor thermal comfort: A field study in Tehran in cold season. Sustainable Cities And Society. 2017;32:153-9.

[71]	Cheung PK, Jim CY. Comparing the cooling effects of a tree and a concrete shelter using PET and UTCI. Building & Environment. 2018;130(feb.):49-61.

[72]	Xu M, Hong B, Jiang R, An L, Zhang T. Outdoor thermal comfort of shaded spaces in an urban park in the cold region of China. Building and Environment. 2019;155:408-20.

[73]	Zoelch T, Rahman MA, Pfleiderer E, Wagner G, Pauleit S. Designing public squares with green infrastructure to optimize human thermal comfort. Building and Environment. 2019;149(FEB.):640-54.

[74]	Lenzholzer S. Research and design for thermal comfort in Dutch urban squares. Resources, conservation and recycling. 2012;64:39-48.

[75]	Middel A, Lukasczyk J, Zakrzewski S, Arnold M, Maciejewski R. Urban form and composition of street canyons: A human-centric big data and deep learning approach. Landscape and Urban Planning. 2019;183:122-32.

[76]	Jin H, Liu S, Kang J. Thermal comfort range and influence factor of urban pedestrian streets in severe cold regions. Energy and Buildings. 2019;198(SEP.):197-206.

[77]	Ali-Toudert F, Mayer H. Numerical study on the effects of aspect ratio and orientation of an urban street canyon on outdoor thermal comfort in hot and dry climate. Building and Environment. 2006;41(2):94-108.

[78]	Li K, Zhang Y, Zhao L. Outdoor thermal comfort and activities in the urban residential community in a humid subtropical area of China. Energy & Buildings. 2016;133(DEC.):498-511.

[79]	Kottek M. World Map of the Kppen-Geiger climate classification updated. Meteorologische Ztschrift. 2006;15(3):259-63.

[80]	National Meteorological Information Centre. 2010–2019. [Available from: <http://data.cma.cn>.

[81]	Concepts R. Thermal environmental conditions for human occupancy. Ansi/ashrae. 2014;5.

[82]	Matzarakis A, Mayer H, Iziomon MG. Applications of a universal thermal index: physiological equivalent temperature. International Journal of Biometeorology. 1999;43(2):76-84.

[83]	Nasrollahi N, Hatami M, Khastar SR, Taleghani M. Numerical evaluation of thermal comfort in traditional courtyards to develop new microclimate design in a hot and dry climate. Sustainable cities and society. 2017;35:449-67.

[84]	Muhaisen AS, Gadi MB. Shading performance of polygonal courtyard forms. Building & Environment. 2006;41(8):1050-9.

[85]	Berkovic S, Yezioro A, Bitan A. Study of thermal comfort in courtyards in a hot arid climate. Solar Energy. 2012;86(5):1173-86.
