# Supplementary material for: Thermal-comfort evaluation of and plan for public space of Maling Village, Henan, China
Source: PLoS One. 2021 Sep 20;16(9):e0256439. doi: 10.1371/journal.pone.0256439 (PMC8452085; doi:10.1371/journal.pone.0256439)
Supplement: Attachment — Submitted filename: Response to Academic Editor.docx [file pone.0256439.s005.docx]

# 1. **Question 1**

Please ensure that your manuscript meets PLOS ONE's style requirements, including those for file naming.

**Response:**

Thank you for your suggestion. We have carefully revised the format of the manuscript in accordance with the requirements of the journal strictly, including those for file naming.

# 2. Question 2 and 5: Ethics statement

**Response:**

The field studies were conducted in Maling village, Changdai Town, Mengjin County, Luoyang City, Henan Province, China. The public open space of the village was taken as the study area. This study was supported by the local government and the masses. The questionnaire survey and field measurement did not involve endangered or protected species. For a detailed explanation, please see the Supporting Information.

# 3. Question 3: Questionnaire investigation

**Response:**

**Outdoor Thermal comfort questionnaire**

Date: /	/____	Time: __________

**1	Gender**

Male Female

Height: _________ Weight: _________

**2	What is your age group range?**

18-29 years

30-40 years

41-50 years

51-60 years

61-64 years

65 years and older

**3	What are you wearing now? (multiple choice)**

Pale colors	Neutral	Dark colors

Upper: Sleeveless vest	Short-sleeve T-shirt

Long- sleeve T-shirt	Long- sleeve blouse	Sweater

Bottom: Short shorts	Straight trousers (thin)	long skirt

Short skirt	Other_______

4 **Location:** Space with fitness facilities	Unsheltered square

Spaces with shading from trees	Unshaded lawns	Green park spaces	Pavilions	Areas with shading from buildings	Roads

**5	Please describe your overall comfort level?**

**（Note:** Please vote according to your actual situation at this time）

**6	How do you feel at this moment?**

**7	Your current activity?**

Standing (chatting, playing mobile phone, enjoy scenery, etc.)

Seating (chatting, playing mobile phone, reading, etc.)

Strolling

Low-intensity exercising (brisk walking, looking after children, walk the dog, etc.)

Medium-intensity exercising (Jogging, etc.)

High- intensity exercising (ball games, square dancing, etc.)

**8	How long have you been here?**

＜15 min	15-30 min	30-60 min	＞60 min

**9	What time do you like to come here during the day?**

6:00-8:00	8:00-10:00	10:00-12:00	12:00-14:00	14:00-16:00	16:00-18:00	18:00-20:00	20:00-21:00

**10	Which meteorological parameters do you think have the greatest impact on thermal comfort?** **(select two of them)**

Air temperature	Relative humidity	Wind speed	Solar radiation

**11	You will be more comfortable if the environment (single choice for each)**

Air temperature: Higher	Unchanged	Lower

Relative humidity: Damper	Unchanged	Drier

Wind speed: Stronger	Unchanged	Weaker

Solar radiation：	Stronger	Unchanged	Weaker

**室外热舒适问卷调查**

日期: /	/____	时间: __________

**1	性别**

男 女

身高: _________ 体重: _________

**2	您处于哪个年龄阶段?**

18-29 岁

30-40 岁

41-50 岁

51-60 岁

61-64 岁

65岁及以上

**3	您现在穿什么? (多选)**

白色	中性色	黑色

**上部:** 无袖背心	短袖T恤	长袖T恤

长袖衬衫	毛衣

**下部:** 超短裤	直裤（薄）	长裙

短裙	其他_______

4**位置:** 有健身设施的地方	无遮挡的广场	遮阳树下

无遮阳的草坪	公园绿地	凉亭

建筑物的阴影区	道路

**5	请描述一下您的整体舒适度？**

（注：请根据本次实际情况投票）

**6	此时此刻你感觉如何?**

**7	您目前的活动?**

站立 (聊天, 玩手机, 欣赏风景等)

坐着 (闲聊, 玩手机, 阅读等)

散步

低强度运动 快步走, 照看孩子, 遛狗等)

中强度运动 (慢跑等)

高强度运动 (球类运动,广场舞等)

**8	您在这里多久了?**

＜15 分钟	15-30 分钟	30-60分钟	＞60分钟

**9	在一天中，您喜欢什么时候来这里？**

6:00-8:00	8:00-10:00	10:00-12:00	12:00-14:00

14:00-16:00	16:00-18:00	18:00-20:00	20:00-21:00

**10	您认为哪些气象参数对热舒适的影响最大？** **(选择其中的两个)**

空气温度	相对湿度	风速	太阳辐射

**11	如果环境是……你会感觉更舒适（每类选择一个）**

空气温度: 更高	不变	更低

相对湿度: 更潮湿	不变	更干

风速: 更强	不变	更弱

太阳辐射：	更强	不变	更弱

# 4. Question 4: Please amend your current ethics statement to include the full name of the ethics committee/institutional review board(s) that approved your specific study

**Response:**

The full name of the institution that approved our research is "people's Government of Changtai Town, Mengjin County".

# 5. Question 6:

The satellite images in Figures 1, 2 and 17 (a) in this study are all from Beijing Qianfan Shijing Co., Ltd, and the contract is signed as follows:

# 6. Question 7:

The ORCID iD of all authors are as follows:

FAN QINDONG, [http://orcid.org/0000-0003-2370-6252](http://orcid.org/0000-0003-2370-6252;)

DU FENGTIAN, <https://orcid.org/0000-0003-4879-2409>

LI HU, <http://orcid.org/0000-0003-3416-8250>

ZHANG CHENMING, https://orcid.org/0000-0001-7579-3285

# 7. Question 8:

We have attached the captions of the supporting information files at the end of the manuscript, and update any in-text citations to match accordingly.

For a detailed explanation, please see the Supporting Information.

# 8. Question 9:

We've removed the images about the participants in the Supporting Information. For a detailed explanation, please see the Supporting Information.
